# Patient-specific *in vitro* follicle development in response to ovarian freezing and culture

**DOI:** 10.1530/RAF-25-0196

**Published:** 2026-07-22

**Authors:** N Kuscu, H Long, K Delray, S Lane, J Davies, C Becker, S A Williams

**Affiliations:** ^1^ Nuffield Department of Women’s & Reproductive Health, University of Oxford, Oxford, United Kingdom; ^2^ Big Data Institute, Department of Statistics, University of Oxford, Oxford, United Kingdom; ^3^ Oxford Cell & Tissue Biobank, Oxford University Hospitals NHS Foundation Trust, Oxford, United Kingdom

**Keywords:** ovary culture, cryopreservation, fertility preservation, paediatric and adolescent girls, follicle development, cancer patients

## Abstract

**Abstract:**

Ovarian tissue cryopreservation is currently the only fertility preservation option for young girls, yet little is known about follicle health and development after freezing in previously treated young cancer patients. This study investigated the effect of cryopreservation on follicle health and developmental potential in ovarian tissue from paediatric and adolescent cancer patients by comparing fresh and cryopreserved tissue from six girls aged 10–16 years, five of whom had received prior cancer treatment. Tissues were cultured *in vitro* for 2 or 8 days, and follicle morphology, health, developmental stage, and density were assessed by histological analysis. Data were analysed using advanced statistical models, including generalized linear mixed models, to account for patient variability. Fresh ovarian tissue contained approximately 15% healthy follicles by Day 8 of culture, whereas cryopreserved tissue showed complete follicular depletion, with 0% healthy follicles remaining. These findings demonstrate the detrimental effects of both the freeze–thaw process and extended *in vitro* culture on follicle health and development in paediatric and adolescent ovarian tissue. Statistical analyses confirmed the significant impact of cryopreservation, as cryopreserved tissue failed to maintain any healthy follicles beyond 1 week in culture, and substantial inter-patient variability was observed (*P* < 0.05), highlighting the influence of individual patient factors. Overall, these findings help resolve discrepancies in the literature regarding follicle survival after ovarian tissue cryopreservation in young cancer patients and underscore the importance of personalized fertility preservation strategies, given the significant inter-patient variability observed. The insights gained offer potential avenues for refining cryopreservation and culture protocols.

**Lay summary:**

Young girls undergoing cancer treatment often rely on ovarian tissue freezing as their only fertility preservation option, yet it remains unclear how well this approach maintains follicle health. Follicles are functional units of the ovary that contain immature eggs and are fundamental to future fertility. In this study, we compared fresh and frozen ovarian tissue from girls aged 10–16 years, most of whom had received chemotherapy, and examined follicle health and developmental potential during *in vitro* culture. Fresh tissue retained a small proportion of healthy follicles after eight days, whereas frozen tissue showed a complete loss of healthy follicles, with considerable differences observed between patients. These findings show that the freeze–thaw process and subsequent culture have a severe impact on follicle health in paediatric and adolescent ovarian tissue. Our work highlights the importance of improving and tailoring fertility preservation methods for young cancer patients and provides important evidence that current approaches to ovarian tissue freezing may not adequately protect follicle health in young cancer patients.

## Introduction

Worldwide, approximately 82,000 prepubertal girls are diagnosed with cancer each year, while in the UK, around 1,900 new cases of childhood cancer are reported annually (https://www.cancerresearchuk.org/health-professional/cancer-statistics/childrens-cancers/incidence#heading-One, Salama *et al.* 2016). Childhood cancer 5-year survival rates now approach 80%, a significant improvement driven by remarkable advancements in therapeutic strategies, such as chemotherapy and radiotherapy, and earlier diagnostic approaches (van den Berg *et al.* 2021). However, such treatments have gonadotoxic potential and can cause ovarian failure and infertility in women (Agarwal & Chang 2007, Coker Appiah *et al.* 2021, Rashidian 2024). Given these improved survival rates and the gonadotoxic effects of cancer treatments, fertility preservation (FP) prior to cancer therapy is recommended (Sugarman & Rosoff 2001, Salama *et al.* 2016, Tarasiewicz *et al.* 2019, Lau & Schaeffer 2020, Dolmans & Donnez 2021).

Becoming a mother after overcoming childhood cancer is also a significant factor in long-term quality of life, which has led to the development of several methods to preserve fertility (Canosa *et al.* 2023). For adult female patients, options such as oocyte cryopreservation, embryo cryopreservation, and ovarian tissue cryopreservation (OTC) are available (Oktay *et al.* 2018, Practice Committee of the American Society for Reproductive Medicine 2019, Ghezelayagh *et al.* 2022). However, young girls and women unable to delay chemotherapy or cannot undergo oocyte collection are limited to OTC as their primary option (Practice Committee of the American Society for Reproductive Medicine 2019, 2021, The ESHRE Guideline Group on Female Fertility Preservation *et al.* 2020). Cryopreserved ovarian tissue can be either autotransplanted or subjected to *in vitro* culture (IVC); however, autotransplantation carries the risk of reintroducing malignant cells, particularly in blood-borne malignancy and ovarian cancer (Dolmans *et al.* 2010, Bastings *et al.* 2013, Dolmans *et al.* 2013). Therefore, OTC and subsequent culture techniques remain the only available options for a specific group of patients. To mitigate this risk, there is a need to develop alternative assisted reproductive technologies, including the use of artificial ovaries and the *in vitro* growth (IVG) of follicles followed by *in vitro* maturation (IVM) and *in vitro* fertilization (Chen *et al.* 2024). A promising strategy for fertility restoration is the *in vitro* activation of dormant primordial follicles through ovarian tissue culture. This process, known as *in vitro* follicle growth (IVFG), enables follicles to transition from a resting state into active growth, offering potential for FP (Ghezelayagh *et al.* 2022). For this purpose, one of the current approaches, IVFG involves a multi-step process that activates dormant primordial follicles in the ovarian cortical tissue, which differentiate into primary follicles and grow into larger secondary follicles. In this approach, preantral follicles are first isolated from ovarian cortical tissue by microdissection and then individually cultured to support further growth. During culture, these follicles can develop into antral follicles with visible fluid-filled cavities. Once they reach the antral stage, the cumulus–oocyte complexes (COCs) are isolated and subjected to IVM to obtain mature oocytes (McLaughlin *et al.* 2018, Telfer 2019).

Sustaining follicle viability and achieving complete oocyte maturation remain major challenges in ovarian tissue culture. Although the multi-step approach has shown promise in producing developmentally competent oocytes using fresh ovarian tissue from healthy donors and a four-step culture protocol (Salama & Woodruff 2019, Telfer 2019), significant challenges remain. Ovarian follicles from cortical biopsies of girls and adolescents have been shown to activate and develop to the secondary follicle stage *in vitro*, demonstrating the developmental competence of immature human ovaries during childhood and adolescence (Anderson *et al.* 2014). However, it remains unclear how translatable these methods are to cryopreserved ovarian tissues from cancer patients, who are often in compromised health and urgently require effective FP strategies. This highlights a critical need to optimize and validate culture protocols specifically for this vulnerable patient group to improve fertility outcomes.

Many patients who could benefit from ovarian tissue culture already have cryopreserved ovarian tissue in storage, and thus, fresh tissue is no longer available. Moreover, it is likely that the biology of these patients, many of whom will have had some level of chemotherapy, may well differ from healthy donors, making the optimization of IVFG for cryopreserved–thawed patient tissues a critical priority (Bjarkadottir *et al.* 2021). Recent studies investigating the effects of freezing, IVC, and grafting on follicle dynamics in frozen adult human ovarian tissue have shown that optimal culture conditions can yield the highest proportion of healthy follicles (Bjarkadottir *et al.* 2021, Hossay *et al.* 2023). While significant progress has been made in FP for women with cancer, despite the high importance, significant knowledge gaps persist for young girls. OTC and ovarian tissue culture are not only essential but also the only available option for FP for certain patient groups, including young girls where their treatment is severely gonadotoxic; however, methods remain experimental. To enhance FP techniques, it is essential to first understand the impact of every stage in the pipeline, including ovarian tissue procurement, processing, cryopreservation, and thawing (Gadek *et al.* 2024). Despite advances in adult ovarian tissue culture, there remains a lack of data on how cryopreserved ovarian tissue from young girls – particularly those who have undergone gonadotoxic treatments such as chemotherapy – responds to IVC.

Therefore, this study aims to fill that gap by improving our understanding of ovarian tissue culture in young cancer patients, specifically by investigating the effects of freezing. Importantly, the study is based on tissue from paediatric and adolescent cancer patients, including those previously exposed to chemotherapy, thereby reflecting the clinical context and complexities associated with FP in this population. The goal is to enhance FP options for this important cohort of cancer patients.

## Materials and methods

### Reagents

Leibovitz’s L-15 medium (11415049), minimum essential medium alpha (αMEM) (22561021), McCoy’s 5A (modified) HEPES-buffered medium (22330021), L-glutamine (25030024), and ascorbic acid (10012011) were purchased from Thermo Fisher. Human serum albumin (HSA) (AI653), ITS liquid media supplement (100×; I3145), penicillin and streptomycin (P0781), sodium pyruvate (S8636), neutral red (N2889), sucrose (S7903), ethylene glycol (324558), Gill no. 2 haematoxylin (GHS232), eosin Y solution (HT110332), and DPX mountant (06522) were purchased from Sigma Aldrich. Recombinant human follicle-stimulating hormone (FSH; Gonal-F; Z1540) and acetic acid (K50116263811) were purchased from Merck Serono (UK), and 24-well culture plates were purchased from Corning Costar (UK). Formalin 10% (1169940) was purchased from VWR.

### Ovarian tissue collection and preparation

The use of human tissue was approved by the Health Research Authority South Central – Oxford B Research Ethics Committee (REC reference: 19/NW/0526). Ovarian cortical tissue was obtained from the Oxford Cell and Tissue Biobank, including both fresh and cryopreserved strips from six paediatric and adolescent patients aged 10–16 years. All were undergoing OTC, as an FP measure due to malignancy. Five of the patients had received chemotherapy or radiation treatment prior to OTC. Permission to use tissue in research had been obtained as part of the consent process. Cortical strips were cryopreserved using slow freezing by the Oxford Cell and Tissue Biobank in 1.5 M ethylene glycol, 0.1 M sucrose, and 10% serum substitute supplement in Leibovitz’s L-15 medium and stored in vapour-phase liquid nitrogen. For clarity, ‘frozen’ tissue is used throughout to refer to samples that were cryopreserved using a controlled freezing protocol to preserve tissue viability.

**Figure 1:**
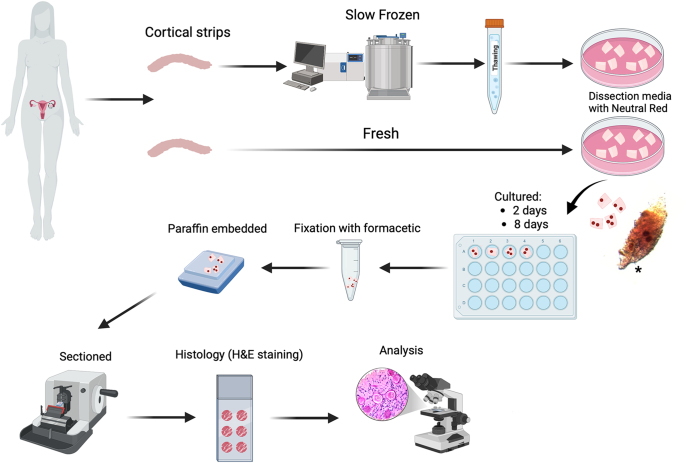
Experimental design. Cortical strips from the same patient (*n* = 6) were collected as both frozen and fresh. Frozen tissues were thawed, while fresh tissues were placed in dissection media with neutral red and cut into small pieces. After incubation, tissue pieces containing follicles were selected for culture. *Representative image collected by N Kuscu. Selected pieces were then transferred to a plate for culturing. On Days 2 and 8, tissues were fixed with Form-Acetic for 24 h, embedded in paraffin, sectioned, and stained with H&E for follicle analysis (created with biorender.com).

The experimental workflow is summarized in Fig. 1.

### Tissue thawing and cortical strip culture

Cortical strips were thawed and processed for culture as described previously (Walker *et al.* 2021). Briefly, following thawing in a water bath (30°C for 3 min), cortical strips were washed through thawing solutions for 5 min each at room temperature to remove cryoprotectants. The thawing solutions contained a reversed ethylene glycol gradient (1.0, 0.5, and 0 M), 0.1 M sucrose, and 3 mg/mL HSA in L-15 medium. Thawed strips were transferred to dissection medium (3 mg/mL HSA, 100 U/mL penicillin, 100 μg/mL streptomycin, 2 mM l-glutamine, and 2 mM sodium pyruvate in L-15 medium) and manually chopped using scalpels and forceps into approximately 0.5 × 0.5 × 0.25 mm pieces. Tissue fragments were incubated in 25 μg/mL neutral red for 1 h to visualize fragments with viable follicles as previously described (Walker *et al.* 2021).

Ovarian cortical pieces from each patient were divided into two groups: fresh and slow frozen/thawed. Tissue pieces were distributed randomly and evenly between different culture conditions (9–12 pieces per condition per patient); fragments, where neutral red staining was observed, were allocated before non-stained fragments. One piece of tissue was placed in each culture well. The tissues were cultured using standard media and protocols as established in our group (Walker *et al.* 2021). The medium used is αMEM, supplemented with 1 mg/mL HSA, 100 U/mL penicillin, 100 μg/mL streptomycin, 2 mM L-glutamine, 10 μg/mL insulin, 5.5 μg/mL transferrin, 5 ng/mL selenium, 50 μg/mL ascorbic acid, and 1 ng/mL recombinant human FSH. Cortical tissue pieces were cultured for either 2 days (*n* = 5) or 8 days (*n* = 6) at 37°C under 5% CO_2_ in air, with medium changes every other day (half the medium removed and fresh medium added). Following the culture period, all tissue pieces were fixed in Form-Acetic 5% solution overnight (Adeniran *et al.* 2021) before storage in 70% ethanol at 4°C.

### Histological analysis

Fixed ovarian tissues were transferred into 70% ethanol and then embedded in Histogel (Epredia™ HistoGel™ Specimen Processing Gel, HG-4000-012, Fisher Scientific, UK) to encapsulate and suspend the small ovarian tissue pieces for easier handling and processing. Then, the tissues were dehydrated in a graded series of ethanol, cleared in xylene, and embedded in paraffin wax.

The wax-embedded tissue was entirely serially sectioned at 5 μm, mounted on glass slides, and stained with haematoxylin and eosin to assess the follicle health and development. Follicle numbers, stages of development, and health status were evaluated. Follicles were staged as previously described (Gougeon 1986, Walker *et al.* 2021) as primordial (single layer of flattened pre-granulosa cells), transitional (single layer with at least one cuboidal granulosa cell), primary (complete layer of cuboidal granulosa cells), and secondary (at least two complete layers of granulosa cells). Follicle health was evaluated morphologically using established criteria (Walker *et al.* 2021), examining granulosa cell pyknosis, oocyte integrity, and ooplasmic shrinkage. We categorized follicles as healthy (with intact oocytes and granulosa cells), non-healthy (showing partial degeneration of either oocytes or granulosa cells), atretic (fully degenerated), or no oocyte follicles (NOFs). Only follicles with a visible nucleus or a clearly defined nuclear membrane were assessed. Follicles were analysed by a researcher in a blinded manner.

### Calculation of follicle density

Follicle density was determined as described (Walker *et al.* 2021) by dividing the total number of follicles counted in a tissue sample by the tissue volume. Every tenth section of each piece of tissue was imaged at a low magnification, and the area was measured using the freehand measuring tool in ImageJ 1.46r (National Institutes of Health, USA; Schneider *et al.* 2012, Rueden *et al.* 2017). Average area measurements were used to calculate the volume of each tissue piece. All imaging was performed using a Leica DM2500 microscope (Leica Microsystems, Germany), fitted with a QImaging Micropublisher 5.0 RTV camera running QCapture Pro 7 software (QImaging, Canada).

### Statistical analysis and modelling

All statistical analyses were performed using R statistical software, version 4.4.2. Descriptive statistics and exploratory visuals were first used to assess follicle density across different experimental groups. One-way ANOVA tests followed by pairwise *t*-tests for multiple group comparisons were used to determine the effect of freezing and culturing on follicle density. To account for intra-patient variability (Walker *et al.* 2021) and tissue volume, a generalized linear mixed model (GLMM) following a Poisson distribution was used to determine the effect of culture conditions on follicle development, as the outcomes were discrete, positive, and right-skewed. To determine the effect of culture conditions on follicle count and density, a linear mixed model, adjusted for patient, was used. Data are presented as mean (±SEM), unless otherwise stated. Statistical significance was defined as *P* < 0.05.

To better account for inter-individual biological variability, patient ID was included as a random intercept in the linear mixed-effects model. We computed the intra-class correlation coefficient (ICC) to quantify the proportion of total variance attributable to patient-level differences:

$$\text{ICC}=\frac{\text{Var}\;\left(\text{patient}\right)}{\text{Var}\;\left(\text{patient}\right)+\text{Residual}\;\text{variance}}=\frac{2.40}{2.40+1.87}\;\approx \;0.56$$

## Results

### Patient population

This study focuses on ovarian tissue from paediatric and adolescent patients aged 10–16 years, a group for whom mature oocyte retrieval is often not feasible or appropriate, particularly in pre- and peri-pubertal stages. While one patient was confirmed to be prepubertal and one postpubertal, the remaining patients were in the early to mid-adolescent range. This variation reflects the real-world clinical diversity in FP candidates within this age group. Samples from six paediatric and adolescent patients (age: 10–16 years, mean: 13.3 ± 2.5 years) were used in this study. Table 1 shows the patients age, diagnosis, and treatment history prior to OTC.

**Table 1:** Patient information and clinical history, including age, diagnosis, and prior treatment history (*n* = 6).

Patient	Age, years	Diagnosis	Treatment prior to OTC
A	10	Osteosarcoma	None
B	11	Relapsed B acute lymphoblastic leukaemia	UK ALL 2011 treatment: 3 g/m^2^ cyclophosphamide; 1,800 mg/m^2^ cytarabine; 75 mg/m^2^ doxorubicin (dates not given in detail)
C	13	Stage IV classic Hodgkin’s lymphoma	Chemotherapy: doxorubicin 80 mg/m^2^ (OEPPA and COPDAC and CED; dates not given in detail)
D	14	Relapsed classical Hodgkin’s lymphoma	Chemotherapy: EuroNet PHL C2 protocol-x2 OEPPA x4 COPDAC pembrolizumab; cyclophosphamide 4,000 mg/2; dacarbazine 3,000 mg/m^2^; doxorubicin 160 mg/m^2^; pembrolizumab 18 mg/kg (EuroNet PHL C2 protocol, OEPPA and COPDAC treatment ended in 2021; pembrolizumab treatment ended in 2023)
E	16	Hodgkin’s lymphoma	ABVD chemotherapy – treatment started in March 2024
F	16	Neurofibromatosis type 1, optic pathway glioma	Vincristine and carboplatin – treatment ended in 2014
			Vinblastine – treatment ended in 2015
			Bevacizumab and irinotecan – treatment ended in 2023
			TPCV treatment prior to OTC (dates not given in detail)

### Cryopreservation impairs follicle health in paediatric and adolescent girls, with progressive decline during long-term culture

**Figure 2:**
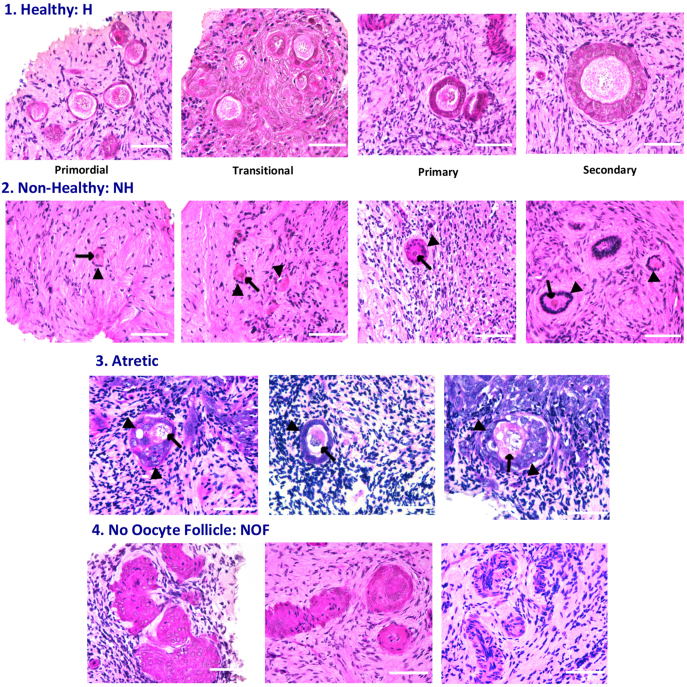
Representative H&E-stained images of follicle classifications. Follicle health was assessed based on the presence or absence of pyknotic granulosa cells (black arrowhead) and pyknotic oocyte/shrunken ooplasm (black arrow). Follicles were categorized into healthy (H), non-healthy (NH), atretic, and no oocyte follicles (NOFs). Both H and NH groups were further subdivided into four subgroups: primordial (single layer of flattened pre-granulosa cells), transitional (single layer with at least one cuboidal granulosa cell), primary (single layer of cuboidal granulosa cells), and secondary (at least two complete layers of granulosa cells). Scale bar: 20 μm.

Follicles were classified into four distinct histological categories: i) healthy, ii) non-healthy, iii) atretic, and iv) no oocyte follicles (NOFs). We observed a group of follicle-like structures and conducted further investigations to confirm their identity. To determine whether these structures were indeed follicles, we performed PAS (Periodic Acid–Schiff) staining, which highlights carbohydrate-rich components in ovarian tissue, including the follicular basement membrane and the zona pellucida surrounding the oocyte, and immunohistochemistry for FOXL2, a granulosa cell marker, and DDX4, an oocyte marker. According to the results, these structures were PAS- and DDX4-negative but FOXL2-positive, indicating that they consisted of granulosa cells without oocytes. In addition to routine analysis of every tenth section, all intervening serial sections (5 µm thickness) were systematically examined in regions of interest during PAS and immunohistochemical analyses. This approach ensured full tissue assessment at high resolution. No oocytes were identified within structures classified as NOFs across these sections, supporting the conclusion that these structures do not represent partially sectioned follicles (Supplementary Fig. 1 (see section on Supplementary materials given at the end of the article)). Therefore, we classified these structures as NOFs. Both healthy and non-healthy follicles were additionally staged by developmental maturity (primordial, transitional, primary, and secondary), as shown in Fig. 2.

**Figure 3:**
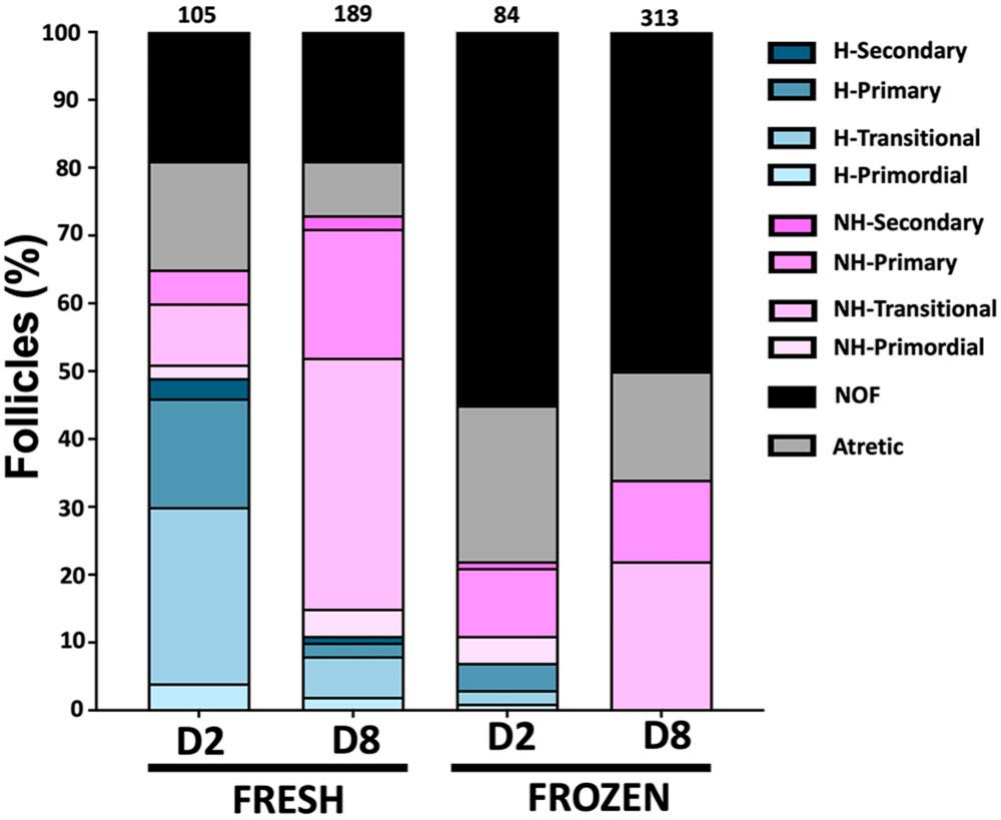
Distribution of follicles (%) in fresh and frozen tissues cultured until Day 2 or Day 8. Shades of blue represent healthy follicles, the lightest blue indicates primordial follicles, and the colour darkens as the follicle grows, with the darkest blue representing growing secondary follicles. Similarly, shades of pink represent non-healthy follicles, grey indicates atretic follicles, and black represents NOFs. Each number displayed above the columns represents the total number of follicles analysed per condition.

The total number of follicles analysed across experimental groups was 105 from fresh tissue (2-day culture), 189 from fresh tissue (8-day culture), 84 from frozen tissue (2-day culture), and 313 from frozen tissue (8-day culture). On Day 2, the fresh group showed a higher proportion of healthy follicles compared to the frozen group (Fig. 3). In fresh tissue at Day 2, healthy follicles comprised 49% of the population, dominated by transitional (26%) and primary (16%) stages, with minor primordial (4%) and secondary (3%) representation. By Day 8, total healthy follicles dropped sharply to 11% (transitional: 6%; primordial/primary: 2% each; secondary: 1%), reflecting a 78% decline, with atretic follicles decreasing to 8% and NOFs stable at 20%. Mirroring this loss, non-healthy transitional follicles exhibited a four-fold increase (from 9 to 37%), suggesting culture conditions adversely affected follicle health and development (Fig. 3).

Cryopreserved tissue showed more severe deterioration, with only 7% healthy follicles at Day 2 (transitional: 2%; primary: 4%; primordial: 1%, and no secondary follicles) alongside increased atresia (23%) and NOFs (56%). By Day 8, healthy follicles were completely absent (0%), atresia persisted at 16%, and NOFs remained high (50%). Notably, the proportion of non-healthy transitional follicles increased, ranging from 0 to 22% (Fig. 3). These findings suggest that freezing negatively impacts follicle health and development.

Overall, fresh tissue retained approximately 15% healthy follicles by Day 8, whereas frozen tissue exhibited complete follicular depletion (0%) (Fig. 3). These results highlight the combined deleterious effects of both freeze–thaw and culture conditions on follicle health and development in paediatric and adolescent girls.

**Figure 4:**
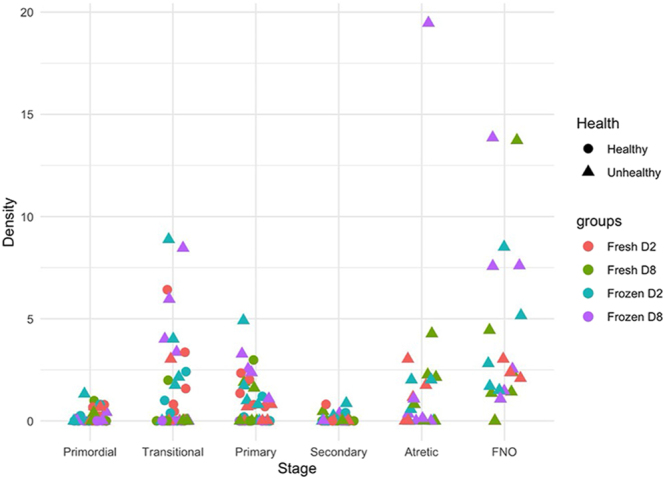
Visualization of follicle density across experimental groups. This graph shows the distribution of follicles based on sample condition (fresh or frozen) and time point (Day 2 or Day 8), with follicle health status also indicated. Groups with higher follicle density are visually distinguishable through the clustering of their corresponding colours and shapes. Each group is represented by a specific colour and shape. Fresh D2 follicles are shown as orange circles, Fresh D8 follicles as green circles, Frozen D2 follicles as turquoise circles, and Frozen D8 follicles as purple circles. Healthy follicles are represented by circles, while unhealthy follicles are represented by triangles.

As outlined in the section titled ‘Materials and methods’, a GLMM was used to assess the effects of culture conditions on follicle development, while adjusting for patient variability and tissue density. Based on this analysis, Fig. 4 illustrates the variation in follicle density distribution across the experimental groups (Fresh D2, Fresh D8, Frozen D2, and Frozen D8), providing an overview of trends in density outcomes. To better account for biological variability, a linear mixed-effects model was fitted with patient as a random effect and fixed effects for freezing status, culture duration, follicle stage, and health status (Fig. 4). Follicle stage was a strong predictor of density (significant in 3 of 5 models). NOFs had higher density compared with atretic follicles (estimate = 1.98, *P* < 0.01), while primordial (estimate = −1.49, *P* = 0.01) and secondary (estimate = −1.59, *P* < 0.01) follicles had lower density. Freezing showed a slight positive effect (estimate = 0.55, *P* = 0.058), and culture duration (D8 vs D2; estimate = 0.03, *P* = 0.93) and follicle health (estimate = 0.40, *P* = 0.213) were not statistically significant. The results revealed an interaction between freezing and follicle health, where unhealthy follicles from frozen tissue had significant increased density (estimate = 1.91, *P* < 0.05).

**Figure 5:**
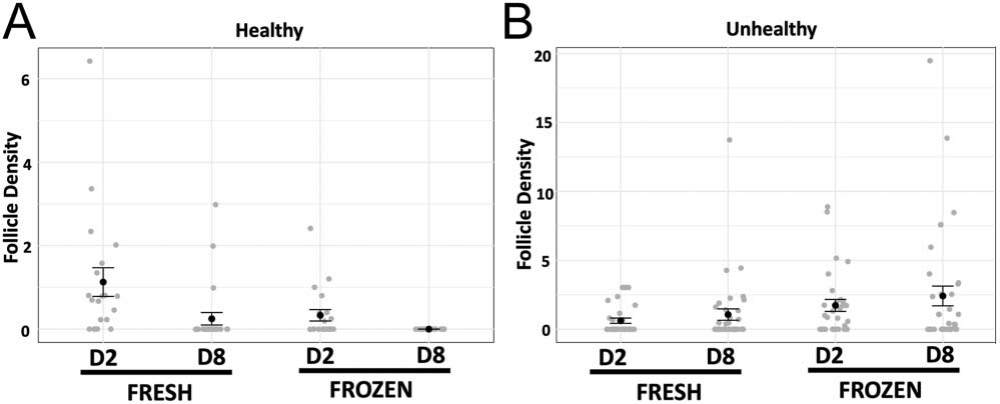
Comparison of healthy (A) and unhealthy (B) follicle density across experimental groups. Graphs represent mean follicle density for each specified state and culture condition (Fresh D2, Fresh D8, Frozen D2, and Frozen D8). The central black dot indicates the mean, with error bars representing the standard error of the mean. The grey dots show individual observations.

When we further analysed follicle density by separating healthy and unhealthy follicles, distinct patterns were observed across the experimental conditions (Fresh D2, Fresh D8, Frozen D2, and Frozen D8). For healthy follicles (Fig. 5A), statistical analysis using one-way ANOVA revealed a significant difference in follicle density between groups (*F*(3, 84) = 6.58, *P* < 0.01). Post hoc pairwise comparisons showed that Fresh D2 had significantly higher density than Fresh D8 (*P* < 0.01), Frozen D2 (*P* = 0.01), and Frozen D8 (*P* < 0.01), suggesting that both freezing and extended culture reduce the density of healthy follicles. In contrast, unhealthy follicles (Fig. 5B) showed only a non-significant trend towards group differences (*F*(3, 128) = 2.50, *P* = 0.063). Among the pairwise comparisons, no difference was observed between Frozen D8 and Fresh D2 (*P* = 0.076), indicating a weaker or more variable response to condition in unhealthy follicles, although this difference was not statistically significant between groups.

These results show that follicle health status modulates the effect of freezing and culturing on follicle density, with healthy follicles being more sensitive to both freezing and prolonged IVC.

**Figure 6:**
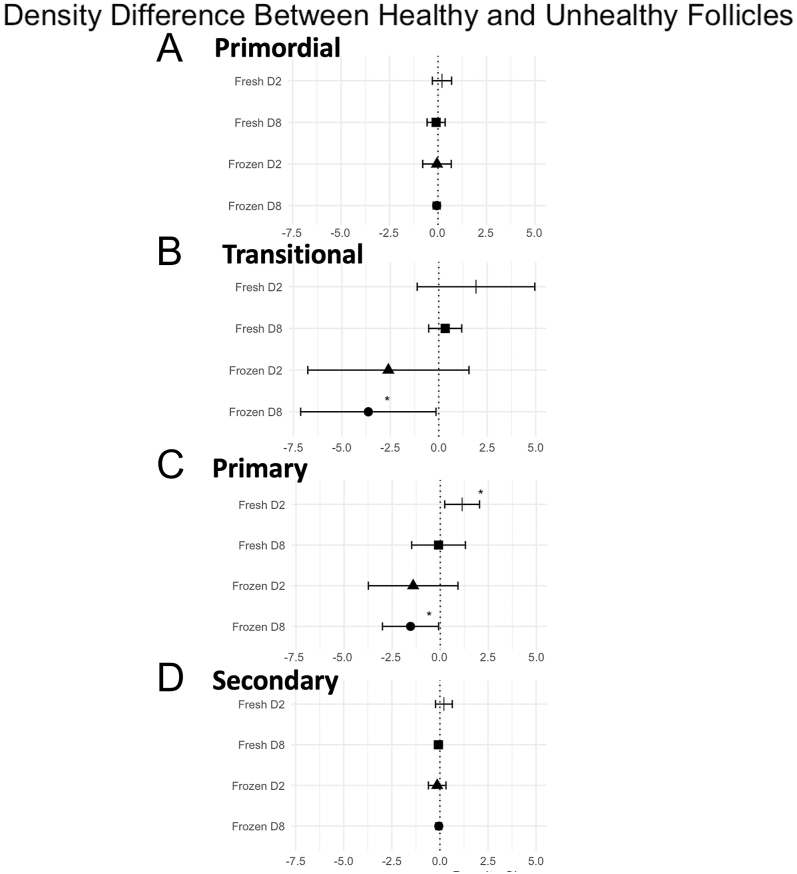
Mixed-effects model for follicle density adjusted by stage, health, and condition. Each of the four graphs represents four distinct developmental stages: (A) primordial follicles, (B) transitional follicles, (C) primary follicles, and (D) secondary follicles. Positive difference = healthy is greater; negative difference = unhealthy is greater. Each point represents the difference in the subset of Fresh D2, Fresh D8, Frozen D2, and Frozen D8 as labelled on the left-hand side. 0 represents no difference in the density. *Significance < 0.01 (significance by pairwise *t*-test).

Furthermore, using a mixed-effects model adjusted for follicle stage, health status, and experimental condition, Fig. 6 shows the difference in average follicle density between healthy and unhealthy follicles across all experimental groups. A key finding from these analyses is that only the primary follicles in the Fresh D2 and Frozen D8 groups, as well as transitional follicles in the Frozen D8 group, showed a significant difference in density between healthy and unhealthy follicles. In all other groups, there is no significant difference in density between healthy and unhealthy follicles. Notably, for both primordial and secondary follicles, there is no evidence of any difference in density change between healthy and unhealthy states. Statistical modelling showed a non-significant main effect of health status on density (*P* = 0.213), but a significant interaction with freezing was observed (*P* < 0.05), suggesting that freezing disproportionately affected density in unhealthy follicles.

Overall, these statistical results support the significant impact of cryopreservation on follicle health and development, with frozen tissue failing to sustain any healthy follicles beyond one week in culture.

### Variability among patients in follicle health and development after cryopreservation

**Figure 7:**
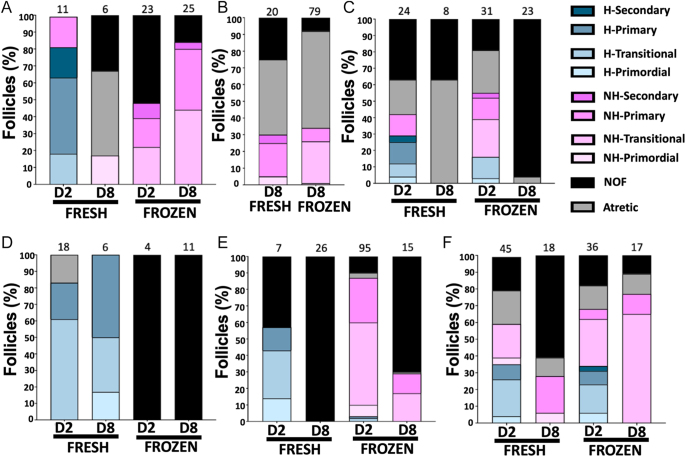
Distribution of follicles (%) in fresh and frozen groups for each patient on Days 2 and 8. Graphs (A)–(E) show data for patients A, B, D, C, E, and F, respectively. Follicles are categorized by colour: shades of blue represent healthy follicles (light blue for primordial follicles; dark blue for growing secondary follicles), pink for non-healthy follicles, grey for atretic follicles, and black for NOFs. Each number displayed above the columns represents the total number of follicles analysed per condition.

To investigate potential correlations or patterns underlying the observed differences in follicle development, individual patient profiles were analysed (Fig. 7). Analyses of six patients revealed substantial inter-patient variability in follicle health patterns across experimental groups.

In patient A’s fresh tissue at Day 2, healthy follicles represented 81%, with developmental stages including transitional (18%), primary (45%), and secondary (18%) follicles, and no atretic follicles or NOFs were detected. By Day 8, healthy follicles were completely depleted (0%), while non-healthy primordial follicles accounted for 17%, alongside atretic follicles (50%) and NOFs (33%). In frozen tissue at Day 2, no healthy follicles were observed (0%), with non-healthy transitional (22%) and primary (17%) follicles present, and a high proportion of NOFs (52%) were observed. By Day 8, non-healthy transitional follicles increased markedly to 44%, with sustained high levels of non-healthy primary follicles (36%), while NOFs decreased sharply to 16% (Fig. 7A). These results indicate that both freezing and extended culture severely reduce healthy follicles, resulting in complete loss of healthy follicles in frozen tissue and progressive degeneration over time in culture.

Analysis of patient B’s Day 8 ovarian tissue demonstrated complete depletion of healthy follicles in both fresh and frozen samples (0%). Fresh tissue exhibited 20% non-healthy primary follicles alongside a high proportion of atretic follicles (45%), whereas frozen tissue showed a notable increase in non-healthy transitional follicles (25%) and pronounced atresia (58%). NOF populations differed substantially between fresh (25%) and frozen (8%) tissue. Notably, only Day 8 cultured tissue was available for analysis for this patient (Fig. 7B).

In patient C’s fresh tissue on Day 2, 29% of follicles were healthy, comprising primordial (4%), transitional (8%), primary (13%), and secondary (4%) stages, alongside 21% atretic follicles and 38% NOFs. By Day 8, healthy follicles were completely depleted (0%), with atresia increasing markedly to 63%. In frozen tissue on Day 2, healthy follicles accounted for 16%, primarily primordial (3%) and transitional (13%) stages, with 26% atretic follicles. By Day 8, atretic follicles declined to 4%, while NOFs comprised 96% of the follicle population (Fig. 7C).

In patient D’s fresh tissue on Day 2, healthy follicles predominated, comprising 83% of the total (transitional 61%, primary 22%), with 17% atretic follicles. By Day 8, the proportion of healthy follicles increased to 100%, distributed as primordial (17%), transitional (33%), and primary (50%) stages. In contrast, frozen tissue exhibited 100% NOFs on both Day 2 and Day 8, with no viable or atretic follicles detected throughout the culture period. These findings indicate that cryopreservation alone significantly reduces follicle viability (Fig. 7D).

In patient E’s fresh tissue on Day 2, 57% of follicles were healthy, comprising primordial (14%), transitional (29%), and primary (14%) stages, with no secondary follicles observed. The remaining 43% were NOFs, with no atretic or non-healthy follicles detected. By Day 8, all follicles had transitioned to NOFs, indicating complete culture-induced degeneration. Frozen tissue on Day 2 exhibited low healthy follicles (3%), limited to transitional (2%) and primary (1%) stages, alongside a high proportion of non-healthy transitional follicles (50%) and moderate NOFs (10%). By Day 8, healthy follicles were absent (0%), with NOFs dominating (70%) and some non-healthy transitional (17%) and primary (12%) follicles remaining (Fig. 7E).

In patient F’s fresh tissue on Day 2, healthy follicles accounted for 35%, comprising primordial (4%), transitional (22%), and primary (9%) stages, with no secondary follicles observed. Atretic follicles and NOFs represented 20%, alongside 20% non-healthy transitional follicles. By Day 8, healthy follicles were completely lost (0%), accompanied by the emergence of non-healthy primary follicles (22%) and NOF dominance (61%). Frozen tissue on Day 2 contained 34% healthy follicles (primordial 6%, transitional 17%, primary 8%, and secondary 3%) with a significant proportion of non-healthy transitional follicles (28%). By Day 8, non-healthy transitional follicles increased markedly to 65%, while NOFs decreased to 12%, indicating complete loss of healthy follicles (0%) (Fig. 7F).

The results from patients C, E, and F demonstrate that both cryopreservation and extended culture conditions lead to complete loss of healthy follicles, with fresh tissue exhibiting progressive atresia and frozen tissue showing a predominant shift towards NOFs.

Altogether, these results reveal that each patient’s profile was different across groups with considerable variability in follicle distribution between individuals. This suggests that patients’ tissues have been affected differently by the freezing and culturing conditions.

As described in the section titled ‘Materials and methods’, a linear mixed-effects model including patient ID as a random intercept was used to account for inter-individual biological variability. The model estimated a variance of 2.40 for the patient random effect and a residual variance of 4.49, indicating substantial variability in baseline follicle density across individuals. The ICC of approximately 0.56 suggests that 56% of the total variance in follicle count is attributable to differences between patients, underscoring the importance of including patients as a random effect in the model.

Overall, these statistical results support that each patient’s profile is significantly different from the other and that there is considerable variability in follicle distribution across patients between groups.

## Discussion

The present findings of our study demonstrate a significant impact of tissue freezing and IVC conditions on follicle health and development in paediatric and adolescent patients, along with significant inter-patient variability. Healthy follicles were particularly sensitive to both cryopreservation and extended culture, with a marked reduction in density observed beyond Day 2, especially in frozen tissue. Notably, the complete absence of healthy follicles in frozen tissue by Day 8 highlights the detrimental effects of prolonged culture following cryopreservation and is a major challenge for FP in paediatric and adolescent girls. A recent study demonstrated that the optimal IVC duration for prepubertal mouse ovaries is 2–4 days, with extended culture leading to marked follicular degeneration (Chen *et al.* 2024). Similarly, Anderson *et al.* (2014) reported that while follicle growth can be initiated in prepubertal human ovarian tissue, progression to later stages is limited and abnormal follicle morphology is more prevalent in younger ovaries. Previous studies have also shown that chemotherapy exposure can adversely affect ovarian tissue quality and follicle survival, leading to increased follicular atresia, altered morphology, and stromal or vascular damage (Asadi Azarbaijani *et al.* 2015, Pampanini *et al.* 2019, Devos *et al.* 2023). These findings highlight the vulnerability of ovarian tissue following gonadotoxic treatment and the importance of optimizing FP strategies for paediatric and adolescent patients. Our results support and extend these observations by demonstrating that prolonged IVC after cryopreservation further compromises follicular integrity and may negatively impact FP outcomes in this cohort.

In this study, we observed that follicle integrity was highly sensitive to both cryopreservation and prolonged IVC, with a marked decline in healthy follicles beyond two days of culture, particularly in frozen tissue. The complete absence of healthy follicles in frozen samples by Day 8 underscores the cumulative detrimental effects of freezing and extended culture on follicle viability. By analysing paired fresh and frozen–thawed ovarian tissue from the same patients over defined culture durations, our study provides new insight into how cryopreservation and culture conditions together shape follicle health and developmental potential in paediatric ovarian tissue. These findings emphasize the need for refined and age- and treatment-specific protocols to improve FP outcomes in young cancer patients.

Statistical analyses further confirmed significant inter-patient variation in follicle density and health, with patient-specific factors accounting for approximately 56% of the total variance. This emphasizes the heterogeneity in tissue characteristics and responses to cryopreservation, independent of the controlled experimental conditions. Precise treatment start and end dates were not available for all patients; therefore, treatment duration and the interval before OTC could not be determined. These factors may partly contribute to the observed variability in follicle health and development. Demographic and clinical factors, such as age, treatment history, and diagnosis, were recorded; however, no consistent associations were observed with follicle outcomes. These results suggest that intrinsic biological differences between young patients may underlie the variability in follicle preservation and viability. Also, our previous study similarly demonstrated wide variation between patients in follicle health and developmental stage within cryopreserved ovarian cortical tissue (Walker *et al.* 2021).

Most of the studies investigate the ovarian function by mapping the cell types in ovarian cortex samples using tissues from, for example, C-delivery patients and transgender and gender-diverse (TGD) patients. However, it has been stated that it is not possible to exclude the potential impact of differing source patient types (FP vs C-delivery and TGD). Factors such as sample sources, sample transport, cryopreservation and storage, quality controls, oocyte culture conditions, tissue dissociation protocols, downstream sample handling, choice of single-cell technique, and bioinformatics strategies can impact the results (Rooda *et al.* 2025). While most previous studies investigating prepubertal ovarian tissue, such as Tsui *et al.* (2023), have focused exclusively on fresh tissue without IVC, they provide valuable quantitative insights into ovarian morphology and follicle density across puberty. Tsui *et al.* reported that prepubertal ovaries exhibit higher primordial follicle density, deeper cortical localization of primordial follicles, and less well-defined subanatomic structures compared with postpubertal ovaries. Although methodological differences prevent direct comparison with our culture-based data, these observations are consistent with the patterns of follicle heterogeneity and density we observed in our samples. By contextualizing our findings against these baseline measurements, we can better interpret the impact of cryopreservation and short-term culture on follicle viability in paediatric ovarian tissue and highlight how intrinsic structural differences established before puberty may influence follicle survival *ex vivo*. Previous studies have reported variable survival rates of adult follicles following cryopreservation; however, there remains a paucity of systematic data evaluating follicle density and viability in young ovarian tissue under culture conditions. Notably, Anderson *et al.* (2014) observed no difference in follicle activation between fresh and frozen tissues; however, their conclusion was based on only two cryopreserved samples from different patients, limiting the robustness of their analysis. In contrast, our study directly compared fresh and frozen tissue from the same patients, thereby eliminating inter-patient variability as a confounding factor. Our findings build on and extend this earlier work by providing a more controlled and clinically relevant evaluation of follicle viability and development in paediatric cancer patients. Importantly, most studies evaluating the impact of cryopreservation and FP techniques on ovarian tissue and consequently on follicle health and development have relied on samples from treatment-naive patients or healthy donors, which may limit the relevance of their findings to tissues previously exposed to chemotherapy or other gonadotoxic treatments. In addition, many studies have caveats in their experimental design, including the use of xenografting ovarian tissue into murine hosts that have not been exposed to the same gonadotoxic therapies as the patients. Considering that alkylating chemotherapy has direct effects on neovascularization and fibrosis within ovarian tissue and likely affects the transplantation site, it is important to take these factors into account to better mimic the clinical conditions experienced by real patients (Gadek *et al.* 2024).

To our knowledge, this study is the first to systematically analyse paired fresh and frozen–thawed ovarian tissue from paediatric and adolescent patients previously exposed to gonadotoxic treatment prior to OTC. The inclusion of this clinically pertinent cohort – which comprised paediatric patients whose ovarian tissues have been cryopreserved and who are likely to require fertility restoration in the near future – provides essential and timely insights into follicle viability and developmental potential. This population represents a uniquely vulnerable group, characterized by a substantially increased risk of fertility impairment consequent to prior gonadotoxic therapy. A comprehensive understanding of follicle health and survival in their cryopreserved tissue is critical not only for advancing fundamental scientific knowledge but also for informing clinical decision-making and optimizing FP protocols. Our results reveal considerable variability in follicle viability across patients, emphasizing the need for further mechanistic studies to elucidate the biological underpinnings of this heterogeneity. Clinically, this variability poses a significant challenge, as cancer survivors currently lack reliable predictive markers to assess the likelihood that their cryopreserved ovarian tissue will successfully restore fertility. Crucially, the design of our study, which employs tissues from the same patients across multiple experimental conditions, reduces patient-related variability and thereby enhances the robustness of our findings. The heterogeneity observed in follicle viability within this cohort highlights the complexity of predicting individual patient outcomes and underscores the imperative for personalized approaches to fertility restoration. Furthermore, these findings establish a vital foundation for the development of refined cryopreservation and transplantation strategies tailored specifically to the unique challenges presented by previously treated patients. Such advancements hold the promise of improving the efficacy and reliability of fertility restoration in this high-risk population.

During a woman’s reproductive lifespan, it is estimated that only approximately 400 oocytes will be ovulated, and even during the peak of fertility, only around 30% of ovulated oocytes are developmentally competent (Rooda *et al.* 2025). This highlights the inherent inefficiency of human follicular development and the rarity of acquiring good-quality, mature oocytes. In contrast, more than 99.9% – and potentially up to 99.99% – of all follicles are destined to undergo atresia, a degenerative process that begins in fetal life and continues throughout adulthood. This natural attrition serves as a major limiting factor for reproductive potential. In our study, following short-term culture of human ovarian tissue for either 2 or 8 days, we observed a high proportion of atretic follicles or follicles lacking an identifiable oocyte (NOF). NOFs were observed across multiple follicular stages, including follicles with multilayered granulosa cell layers but no detectable oocyte, suggesting that oocyte loss or degeneration may occur while granulosa cells remain transiently preserved prior to complete follicular regression. This supports the interpretation that NOFs may represent an intermediate stage of follicular degeneration rather than a distinct follicle type or a culture artefact. These findings are consistent with the expected biological pattern of follicle loss *in vivo* and suggest that many of the follicles present at the start of culture may have already been predisposed to atresia. Given that the overwhelming majority of follicles are destined for degeneration, it remains unclear whether any individual follicle observed *ex vivo* would have progressed to full maturation *in vivo*. Furthermore, the presence of NOFs in both fresh and cryopreserved tissue suggests that they are unlikely to arise solely as a consequence of culture or cryopreservation, but may also reflect intrinsic follicular heterogeneity and physiological degenerative processes within the ovarian reserve. The observed increase in NOFs following cryopreservation and extended culture likely reflects the combined effects of this intrinsic heterogeneity and increased susceptibility of follicles to cellular stress induced by chemotherapy exposure, freezing–thawing, and IVC conditions. Recent single-cell studies have further highlighted substantial interfollicular heterogeneity within human ovarian tissue, identifying transcriptionally distinct follicle populations with differing molecular profiles and stress responses (Rooda *et al.* 2025). Although NOFs were not specifically defined in that study, these findings support the concept that follicles within the ovarian cortex exist along a continuum of developmental competence and degenerative susceptibility, which may underlie the NOF phenotype observed in this study.

A more detailed understanding of follicular atresia, including early morphological markers such as NOFs, could, therefore, contribute to improving FP strategies. This includes refining methods to distinguish viable follicles from those committed to degeneration and developing approaches to support follicular survival and growth *in vitro*.

Despite advances in OTC techniques, tissue damage remains an unavoidable challenge due to the heterogeneous cellular composition of the ovary. However, freezing and thawing of cells and tissues is a damaging process, and so the protocols used must be tightly controlled to minimize this damage. Cooling rate, ice nucleation temperature, cryoprotectant agent (CPA) used, and storage conditions are key factors. These optimal parameters differ between different cell types, functions, and biological structures and so must be characterized for each biological system. One of the major sources of injury during freezing and thawing is ice crystal formation, which can physically disrupt follicular structures and stromal integrity. Cryopreserved ovarian tissues are also highly susceptible to oxidative stress and apoptosis, contributing further to follicular loss and stromal cell death. Excessive production of reactive oxygen species (ROS) during cryopreservation negatively affects reproductive outcomes, including reduced embryo development and potential health issues in progeny, despite ROS being a normal component of cellular physiology (Zhang *et al.* 2023). When researchers started to carry out immature ovary cryopreservation, the freezing and thawing was applied from adult protocols and was not specifically optimized for the immature ovaries. Importantly, the ovary undergoes significant physiological and structural changes with age. Paediatric and adolescent ovaries differ from adult ovaries in follicle density, stromal composition, and metabolic activity. It has been shown that prepubertal and pubertal ovaries differ significantly from adults: prepubertal ovaries contained a high proportion of morphologically abnormal non-growing follicles and their follicles demonstrated reduced IVG potential compared to adult tissue (Anderson *et al.* 2014). These differences imply that cryopreservation protocols optimized for adult ovarian tissue may not be fully effective or safe for young ovarian tissue. For instance, variations in tissue water content and follicle developmental stage may influence susceptibility to ice crystal injury and oxidative damage. Therefore, freezing and thawing protocols must be specifically optimized for the unique characteristics of paediatric and adolescent ovarian tissue. Refining cryoprotectant formulations, cooling and warming rates, and antioxidant supplementation tailored to the paediatric ovary is essential to maximize follicle viability and developmental potential. As a result, there is significant room to develop and validate age-specific protocols that reduce cryoinjury and improve clinical FP outcomes for children and young cancer patients.

In conclusion, our results underscore the urgent need to refine FP techniques to achieve consistent and reproducible outcomes for young cancer patients. This study provides important insight into the impact of cryopreservation and short-term culture on follicle health; however, comprehensive molecular analyses – such as transcriptomic profiling, gene expression studies, and epigenetic assessments – have not yet been conducted. Incorporating such analyses in future studies would enable a more in-depth evaluation of follicle development and the cellular mechanisms underlying follicle viability post-cryopreservation. These findings provide a scientific framework to support ongoing efforts to optimize FP strategies for young cancer patients undergoing gonadotoxic treatments, with the ultimate aim of improving reproductive outcomes in this vulnerable population. Continued prioritization of tailored approaches for different patient groups, particularly for young girls who have already stored ovarian tissue and may require fertility restoration in the future, will be crucial for advancing FP success.

## Supplementary materials



## Declaration of interest

The authors declare that no conflict of interest could be perceived as prejudicing the impartiality of the research reported.

## Funding

This project was made possible in part by grant number 2021-238038 from the Chan Zuckerberg Initiative DAF, an advised fund of Silicon Valley Community Foundation, to SAW to fund NK. This project was supported by the EPSRC Centre for Doctoral Training in Healthcare Data Science grit (EP/Y035321/1) awarded to KD.

## Author contribution statement

NK and SW designed the study. NK performed the experiments, collected the data, and analysed and interpreted the results. HL and KM conducted detailed statistical analyses. SL, JD, and CB provided the ovarian tissue. NK wrote the original manuscript and SW critically revised it. All authors approved the final version of the manuscript.

## Data availability

The data of this study are available from the corresponding author upon reasonable request.
