# Supplementary figures and images for: Patient-specific in vitro follicle development in response to ovarian freezing and culture
Source: Reprod Fertil. 2026 Jul 22;7(3):RAF250196. doi: 10.1530/RAF-25-0196 (PMC13393308; doi:10.1530/RAF-25-0196)

**H&E**

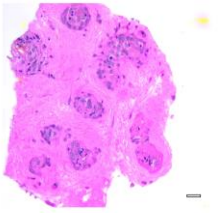

**PAS**

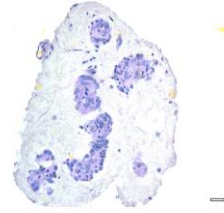

**DDX4**

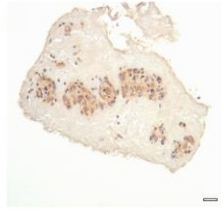

**FOXL2**

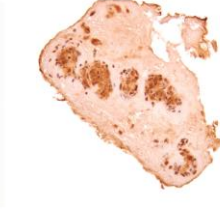

**Negative Control**

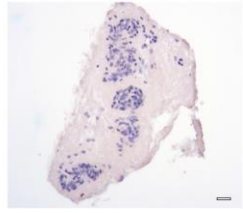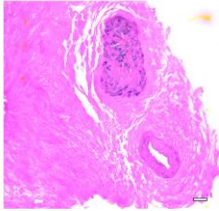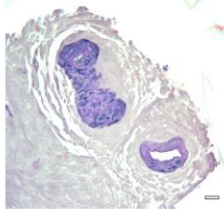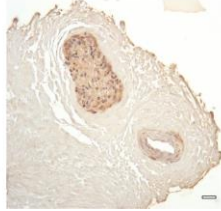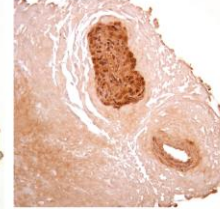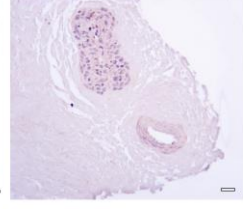

Supplement: Supplementary file 1 [file RAF-25-0196_supplementary_figure_1.pdf]
